# Multi-species data integration and gene ranking enrich significant results in an alcoholism genome-wide association study

**DOI:** 10.1186/1471-2164-13-S8-S16

**Published:** 2012-12-17

**Authors:** Zhongming Zhao, An-Yuan Guo, Edwin JCG van den Oord, Fazil Aliev, Peilin Jia, Howard J Edenberg, Brien P Riley, Danielle M Dick, Jill C Bettinger, Andrew G Davies, Michael S Grotewiel, Marc A Schuckit, Arpana Agrawal, John Kramer, John I Nurnberger, Kenneth S Kendler, Bradley T Webb, Michael F Miles

**Affiliations:** 1 Department of Biomedical Informatics, Vanderbilt University School of Medicine, Nashville, TN 37232, USA; 2 Department of Psychiatry, Vanderbilt University School of Medicine, Nashville, TN 37232, USA; 3 Department of Cancer Biology, Vanderbilt University School of Medicine, Nashville, TN 37232, USA; 4 Key Laboratory of Molecular Biophysics of the Ministry of Education, College of Life Science and Technology, Huazhong University of Science and Technology, Wuhan 430074, China; 5 Center for Biomarker Research and Personalized Medicine, School of Pharmacy, Medical College of Virginia, Virginia Commonwealth University, Richmond, VA 23298, USA; 6 Virginia Institute for Psychiatric and Behavioral Genetics, Virginia Commonwealth University, Richmond, VA 23298, USA; 7 Department of Biochemistry and Molecular Biology, Indiana University School of Medicine, Indianapolis, IN 46202, USA; 8 Department of Human and Molecular Genetics, Virginia Commonwealth University, Richmond, VA 23298, USA; 9 Department of Pharmacology and Toxicology, Virginia Commonwealth University, Richmond, VA 23298, USA; 10 Department of Psychiatry, University of California, San Diego, La Jolla, CA 92093, USA; 11 Department of Psychiatry, Washington University School of Medicine, Saint Louis, MO 63110, USA; 12 Department of Psychiatry, University of Iowa College of Medicine, Iowa City, IA 52242, USA; 13 Institute of Psychiatric Research, Department of Psychiatry, Indiana University School of Medicine, Indianapolis, IN 46202, USA

## Abstract

**Background:**

A variety of species and experimental designs have been used to study genetic influences on alcohol dependence, ethanol response, and related traits. Integration of these heterogeneous data can be used to produce a ranked target gene list for additional investigation.

**Results:**

In this study, we performed a unique multi-species evidence-based data integration using three microarray experiments in mice or humans that generated an initial alcohol dependence (AD) related genes list, human linkage and association results, and gene sets implicated in *C. elegans *and *Drosophila*. We then used permutation and false discovery rate (FDR) analyses on the genome-wide association studies (GWAS) dataset from the Collaborative Study on the Genetics of Alcoholism (COGA) to evaluate the ranking results and weighting matrices. We found one weighting score matrix could increase FDR based q-values for a list of 47 genes with a score greater than 2. Our follow up functional enrichment tests revealed these genes were primarily involved in brain responses to ethanol and neural adaptations occurring with alcoholism.

**Conclusions:**

These results, along with our experimental validation of specific genes in mice, *C. elegans *and *Drosophila*, suggest that a cross-species evidence-based approach is useful to identify candidate genes contributing to alcoholism.

## Background

Research on the genetics and neurobiology of alcoholism uses a variety of study designs and model organisms. A wealth of data are available, including linkage studies in human alcoholics, microarray studies of inbred mouse strains' brains and rat brains exposed to ethanol, and studies of loss or gain of function of genes in organisms such as *C. elegans *and *Drosophila *[1,2]. Although results or information across experiments are often compared by individual researchers in order to generate hypotheses, interpret results, or prioritize targets for follow up investigations [3], these analyses are not always done comprehensively and rarely include a cross-species approach [4-7]. While data integration itself can be challenging, how best to utilize combined results is also unclear. Although pooled results may yield valuable insights, there are potential benefits of using more systematic approaches to generate quantitative rankings that can then, in turn, guide additional studies. In particular, these rankings could be applied to choosing molecular targets for knockdown studies in model organisms or genetic association studies in humans. For this and other approaches, evaluation is often needed in order to determine whether the rankings are effective at the end of the data integration process.

Challenges exist for each stage of an integration process, including the creation of an empirical gene list across species and platforms, scoring the information, and then evaluating the scoring system itself. For example, once various data are collected, identifying the best way to integrate them poses a problem since the criteria for selecting gene lists often differ substantially across studies. Specifically in microarray studies, the expression of gene-specific transcripts is selected via statistical threshold(s), but individual genes can have multiple transcripts that may differ in their abundance [8]. Therefore, a given gene can yield multiple expression values through microarray or next generation RNA sequencing (RNA-Seq) analyses. Likewise, human genetic association studies test multiple genetic markers, usually single nucleotide polymorphisms (SNPs), across a gene. In contrast, the results of genetic linkage or quantitative trait locus (QTL) studies in humans or mice can span tens of megabases and contain potentially hundreds of genes. Furthermore, low replication rates and identification of non-functional markers in most studies makes the search for true genetic signals difficult [9-11]. While there are issues with data reduction or summarization, integration at the level of the gene can be used as a link across a number of commonly used approaches.

If genetic information is summarized at the gene level, then each gene in the genome can be assigned a score for each experiment or data set available. This measurement can be quantitative or qualitative. For example, p-values may be assigned to a gene within a quantitative trait locus (QTL) or a linkage region. However, differences in gene-specific p-values within an interval of interest may be misleading since linkage peaks can shift, and variants responsible for the linkage may not be at the peak itself. In contrast, large numbers of genes may show altered expression in microarray studies and represent real changes due to signal cascades affecting entire gene networks. These correlated expression networks, in which a large number of changes are expected, contrast with linkage regions, in which most if not all genes do not actually contain variant(s) linked to the disease. A combined p-value method can be used for quantitative analyses, but this approach may present its own challenges. The individual data sources may not be weighted equally since the relative magnitudes of the p-values can be vastly different across platforms (e.g., mouse and human QTL studies). To avoid such issues, qualitative scores that measure the presence or absence of evidence above a threshold may be used, but thresholds have their own problems. Regardless of scoring choice, and despite some problems associated with each, a combined gene rank score can be generated from data integration. These gene rank scores can be used to perform weighted analysis or to define gene subsets for further investigation.

The effectiveness of such ranking can be verified by conducting further testing on genes ranked highly in the analyses. Alternatively, because the design of genome-wide association studies (GWAS) is hypothesis free, this approach offers opportunities to empirically test a ranking method and provide insight into further refinement, and all or most potential candidate genes can be tested in one experiment. If higher ranked genes contain more significant SNPs than a random set of genes, then the utility of a cross-species and platform integration and ranking approach would be demonstrated [12]. In this report, we attempt to implement and evaluate the utility of the approach outlined above by collecting data across species and approaches, summarizing at the gene level, ranking the genes, and testing the rankings in complex traits related to alcoholism and ethanol response. We included data generated from ethanol response experiments because this trait is one of the contributing factors for alcoholism [13].

## Results

### Ranked gene list

**Figure 1:**
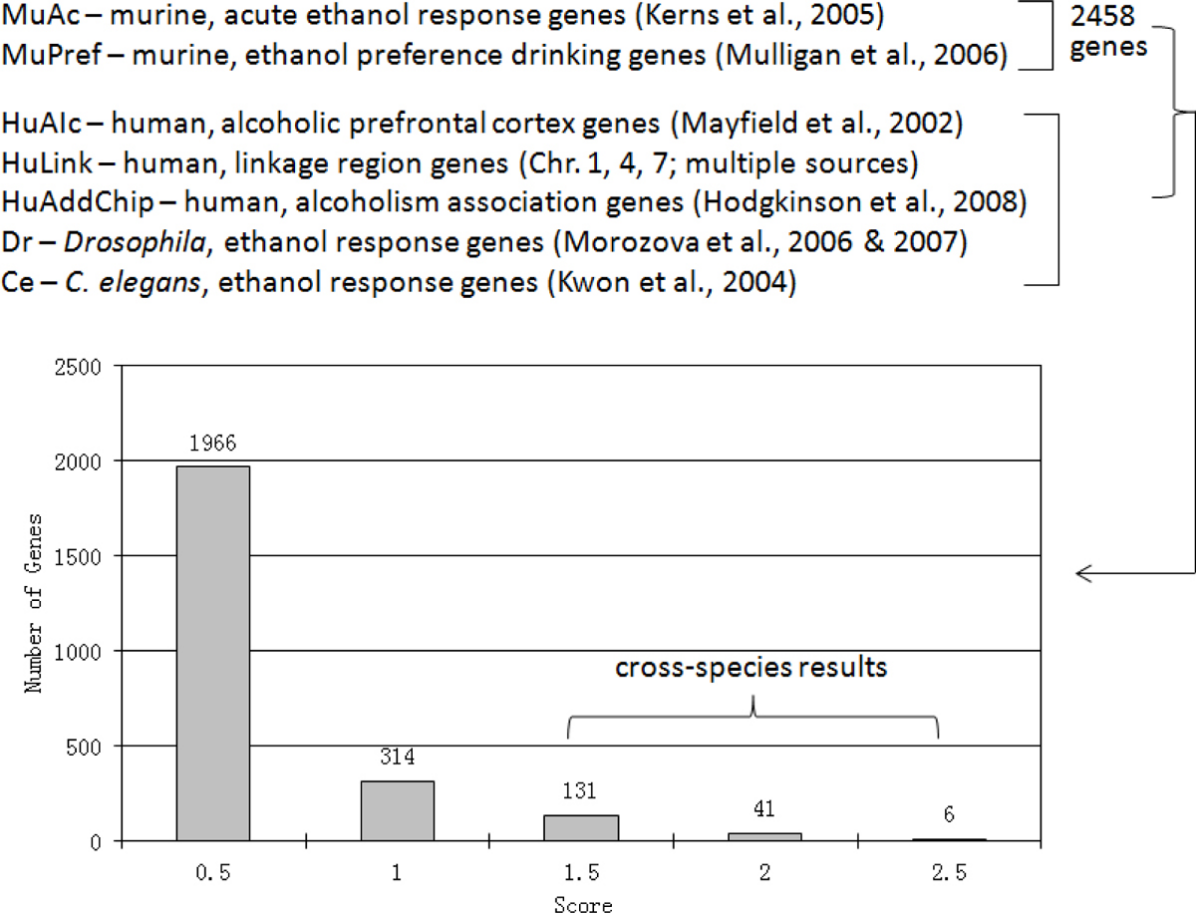
**Data sources and ranking score results using weighting score matrix 3**. The details of weighting score matrix 3 are provided in Table 1.

An initial list of 2458 genes that show altered expression in mouse brain in response to ethanol in two previous studies [3,14] was used as a starting point. These datasets were abbreviated as MuAc and MuPref (see Figure 1). Five additional data sources were used to construct a score for these genes, including 1) genes showing altered expression in the prefrontal cortex of human alcoholics (abbreviated as HuAlc) [15], 2) linkage intervals from published studies of the Collaborative Study on the Genetics of Alcoholism (COGA) and the Irish Study of Alcoholism samples (abbreviated as HuLink) [16-18], 3) genes contained on a human addiction/alcoholism array (abbreviated as HuAddChip) [19], 4) those from a smaller list of ethanol-related genes compiled from *Drosophila *(abbreviated as Dr) [20,21], and 5) a short list of ethanol-related genes compiled from *C. elegans *(abbreviated as Ce) [22]. Additionally, genes having cross-species hits acquired bonus scores (the "Cross" score in our algorithm, see Table 1), as cross-species evidence was regarded as an important factor in gene salience. Here, we used score to estimate the evidence of a gene, rather than using a quantitative measurement (e.g., significance level, see section Materials and methods). We proposed 10 weighting score matrices (Table 1). The corresponding ranking results are shown in Table 2.

**Table 1:** Ten weighting score matrices used in cross-species data integration and gene ranking.

Weighting score matrix (WSM)	Description
WSM1: (0.5,0.5,1,1,0.5,0.5,0.5,1) *	HuAlc, HuAddChip, and Cross ** given 1, all others 0.5
WSM2: (0.5,0.5,1,1,0.5,0.5,0.5,0.5)	HuAlc and HuAddChip given 1, all others 0.5
WSM3: (0.5,0.5,0.5,1,0.5,0.5,0.5,0.5)	HuAddChip given 1, all others 0.5
WSM4: (0.5,0.5,1,1,1,0.5,0.5,0.5)	All human data given 1, all others 0.5
WSM5: (1,1,1,1,0.5,0.5,0.5,0.5)	All mouse data, human HuAlc and HuAddChip given 1, all others 0.5
WSM6: (1,1,0.5,0.5,0.5,0.5,0.5,0.5)	All mouse data given 1, all others 0.5
WSM7: (1,1,1,1,1,0.5,0.5,0.5)	All mouse and human data given 1, all others 0.5
WSM8: (0.5,0.5,0.5,1,0.5,0.5,0.5,1)	HuAddChip and Cross given 1, all others 0.5
WSM9: (1,1,1,1,1,1,1,1)	All given 1
WSM10: (1,1,1,1,1,1,1,0.5)	All but Cross given 1

* The weights in the parentheses are in the order for the datasets: MuAc, MuPref, HuAlc, HuAddChip, HuLink, Ce, Dr, Cross, respective. The description of these datasets is provided in Figure 1.

** Cross denotes the bonus score for genes who had cross-species hits except for genes from human linkage regions (see section Materials and methods).

**Table 2:** Summary of the genes by their scores using 10 weighting matrices*.

Weighting matrix	Score							
	
	0.5	1	1.5	2	2.5	3	3.5	4
1	1722	535	40	38	102	21		
2	1722	535	78	102	21			
**3**	**1966**	**314**	**131**	**41**	**6**			
4	1722	338	235	137	16	10		
5		1966	220	137	112	15	8	
6	244	1745	202	222	34	11		
7		1966		357	89	29	17	
8	1966	314	17	114	41	6		
9		1966		314		149		29
10		1966		314	132	17	29	

* For each matrix, scores of each gene were summarized based on its evidence in each dataset (see details in Table 1). Then, the number of genes in each score range was counted and summarized in this table.

### Alcohol dependence GWAS analysis for ranked genes

To assess the performance of our ranking algorithm and weighting score matrices, we explored whether the ranked genes showed non-random enrichment of significant signals in alcoholism GWAS results. Specifically, we examined enriched association signals of ranked genes in the COGA GWAS [23], one of the largest alcohol dependence GWAS datasets. To increase the effect of our analyses we filtered the data for minor allele frequency, Hardy-Weinberg equilibrium deviation, and failure rate (see section Materials and methods). This resulted in 958,380 SNPs in our follow up analysis, with an observed minimum p-value of 9.5 × 10^-7^. However, the minimum q-value was 0.605 after False Discovery Rate (FDR) analysis. Of those SNPs approximately 68.7% (658,008/958,380) mapped to the human non-pseudogenes in NCBI Entrez Gene database, and they were used in this study. FDR analysis was then performed on restricted subsets of markers based on gene rank score.

For each ranked gene under different weighting score matrices, we calculated the q-value of each SNP in the gene from the COGA GWAS data. The results for the ten weighting score matrices were summarized in Additional file 1. For each weighting score matrix, its gene ranking performance was expected to increase by improving the q-values of SNPs mapped in the ranked genes. To quantitatively measure performance and correct for gene size, we conducted 100 simulations, in each of which the same number of genes were randomly chosen from the whole gene set. FDR based q-value analysis was then performed on the GWAS genotyped SNPs that mapped to the randomly chosen genes. The proportion of q-values in each q-value bin (e.g., 0.1-0.2) was calculated and then compared with those from the actual ranked alcohol genes. For the simplicity of comparison, we separated q-values into different bins. The results are shown in Additional file 1.

According to our permutation results, the weighting score matrix 3 had the best performance, since it gave the lowest q-values among genes (Additional file 1). This matrix was then used to refine gene scores using 1000 permutations (Table 3). In general, the subset of SNP results restricted to the scored genes was enriched for significant effects as the gene rank score increased from 0.5 to 2.0 (see Table 4). Specifically, the minimum FDR based q-value was 0.605 for all SNPs passing QC. The minimum q-value decreased for SNPs in all scored genes, but then increased for genes with score ≥ 1 or ≥ 1.5. However, the minimum q-value became the smallest (0.357) when this analysis was applied to genes with score ≥ 2. There were 47 genes whose scores were ≥ 2, and a total of 2293 SNPs mapped to these genes. For this gene subset, we found many more SNPs having small q values, including 27 SNPs with q-value < 0.4 and 39 SNPs with q-value < 0.5, than those in other gene sets (e.g., gene subset with score ≥ 1.5 or any scored, Table 4). Although this q-value analysis was not perfect (e.g., we did not find a steady decrease of q-value by increasing gene score, Table 4), it suggests that multi-species gene ranking by optimal weighting matrix might be effective for prioritizing candidate genes for complex traits.

**Table 3:** Empirical p-values estimated from 1000 permutations based on weighting score matrix 3.

q-value	Gene score				
	
	≥0.5	≥1	≥1.5	≥2	= 2.5
q < 0.9	0.393 (6399)	0.367 (1863)	0.603 (399)	0.191 (866)	0.052 (178)
q < 0.8	0.525 (469)	0.292 (415)	0.441 (163)	0.310 (199)	0.131 (108)
q < 0.7	0.495 (8)	0.228 (164)	0.627 (5)	0.208 (117)	0.118 (72)
q < 0.6	0.286 (5)	N/A	N/A	0.225 (42)	0.093 (53)
q < 0.5	0.113 (5)	N/A	N/A	0.122 (39)	N/A
q < 0.4	0.012 (5)	N/A	N/A	0.101 (27)	N/A

Based on Table 2, we used weight matrix 3 to rank genes (a total of 2458 genes) and then separated them according to their q-values. The number of SNPs in each q-value and score category based on COGA dataset is shown in parentheses. N/A: not available due to absence of the real data at those categories.

**Table 4:** Improvement in GWAS-based association FDR as multi-species gene ranking score increases.

FDR q-value	Total SNPs passing QC	Scored	Score ≥1	Score ≥1.5	Score ≥2	Score = 2.5
All SNPs	547920	91774	18988	7948	2293	210
< 0.9	48016	6399	1863	399	866	178
< 0.8	643	469	415	163	199	108
< 0.7	6	8	164	5	117	72
< 0.6	0	5	0	0	42	53
< 0.5	0	5	0	0	39	0
< 0.4	0	5	0	0	27	0
< 0.3	0	0	0	0	0	0
< 0.2	0	0	0	0	0	0
< 0.1	0	0	0	0	0	0
< 0.05	0	0	0	0	0	0
Min q	0.605	0.369	0.667	0.636	0.357	0.526

### Bioinformatics analysis of top ranked genes

As presented above and detailed in Table 5, 47 genes with score ≥ 2 had promising q-value improvement, and were used as our high priority list for follow up bioinformatics analysis. These genes also had evidence from at least 2 different species (Figure 1). We first performed functional enrichment analysis of Gene Ontology (GO) terms implemented in the WebGestalt tool. In this tool, each gene set is tested its functional enrichment with GO annotations based on the hypergeometric test. As shown in Table 6 the most significantly enriched functional terms belonged to the groupings of neurotransmitter receptor activity, ion binding, and synaptic structure. The most significantly enriched functional terms were behavior (p_BH _= 5.08 × 10^-5^), gamma-aminobutyric acid (GABA) signaling pathway (p_BH _= 8.51 × 10^-5^) and cell communication (p_BH _= 0.0001) in the GO category of "Biological Process"; GABA receptor activity (p_BH _= 3.35 × 10^-9^), GABA-A receptor activity (p_BH _= 7.38 × 10^-8^), and neurotransmitter binding (p_BH _= 7.38 × 10^-8^) in the category of "Molecular Function"; and postsynaptic membrane (p_BH _= 1.86 × 10^-7^), chloride channel complex (p_BH _= 2.76 × 10^-7^), and synapse part (p_BH _= 3.36 × 10^-7^) in the category of "Cellular Component". Many of these enriched functional categories are consistent with the current knowledge of alcohol dependence and ethanol response [24]. These indicate that the top ranked genes are highly enriched in functions relevant to alcoholism.

**Table 5:** 47 genes with ranked score ≥ 2 using weighting score matrix 3.

Gene symbol	Gene ID	Score
*TAC1*	6863	2.5
*JUN*	3725	2.5
*GABRB1*	2560	2.5
*GABRA2*	2555	2.5
*CCKBR*	887	2.5
*CCK*	885	2.5
*TMEM165*	55858	2
*TIMP2*	7077	2
*TH*	7054	2
*SPP1*	6696	2
*SLC6A11*	6538	2
*PURB*	5814	2
*PRKCE*	5581	2
*PPP1R1B*	84152	2
*PENK*	5179	2
*PDZRN3*	23024	2
*PDAP1*	11333	2
*PC*	5091	2
*PBX1*	5087	2
*NTSR2*	23620	2
*NTRK2*	4915	2
*NR4A3*	8013	2
*NPY*	4852	2
*NPY2R*	4887	2
*MPDZ*	8777	2
*MAPK14*	1432	2
*MAN1A2*	10905	2
*LAMB1*	3912	2
*GSK3B*	2932	2
*GPRC5B*	51704	2
*GNG12*	55970	2
*GBA*	2629	2
*GABRG2*	2566	2
*GABRD*	2563	2
*GABRB2*	2561	2
*GABBR1*	2550	2
*FOXN3*	1112	2
*FOSL2*	2355	2
*EIF4EBP2*	1979	2
*DDX5*	1655	2
*CLIC4*	25932	2
*CHRM1*	1128	2
*CAPZB*	832	2
*BDNF*	627	2
*ATP8A1*	10396	2
*ASNS*	440	2
*ABHD4*	63874	2

**Table 6:** Functional enrichment test for the 47 top ranked genes using WebGestalt.

GO ID*	Term	# genes	p-value	p_BH_**
GO:0007610 (BP)	Behavior	11	1.17 × 10^-7^	5.08 × 10^-5^
GO:0007214 (BP)	Gamma-aminobutyric acid signaling pathway	4	3.92 × 10^-7^	8.51 × 10^-5^
GO:0007154 (BP)	Cell communication	29	7.66 × 10^-7^	0.0001
GO:0007631 (BP)	Feeding behavior	5	2.16 × 10^-6^	0.0002
GO:0050794 (BP)	Regulation of cellular process	37	3.00 × 10^-6^	0.0002
GO:0050789 (BP)	Regulation of biological process	38	1.73 × 10^-6^	0.0002
GO:0033555 (BP)	Multicellular organismal response to stress	4	3.21 × 10^-6^	0.0002
GO:0007186 (BP)	G-protein coupled receptor protein signaling pathway	14	4.88 × 10^-6^	0.0002
GO:0007166 (BP)	Cell surface receptor linked signal transduction	19	2.53 × 10^-6^	0.0002
GO:0065007 (BP)	Biological regulation	38	8.70 × 10^-6^	0.0003
GO:0016917 (MF)	GABA receptor activity	6	3.22 × 10^-11^	3.35 × 10^-9^
GO:0004890 (MF)	GABA-A receptor activity	5	1.93 × 10^-9^	7.38 × 10^-8^
GO:0042165 (MF)	Neurotransmitter binding	7	2.13 × 10^-9^	7.38 × 10^-8^
GO:0030594 (MF)	Neurotransmitter receptor activity	6	3.56 × 10^-8^	9.26 × 10^-7^
GO:0005254 (MF)	Chloride channel activity	6	6.04 × 10^-8^	1.26 × 10^-6^
GO:0005253 (MF)	Anion channel activity	6	9.06 × 10^-8^	1.35 × 10^-6^
GO:0031404 (MF)	Chloride ion binding	6	9.06 × 10^-8^	1.35 × 10^-6^
GO:0043168 (MF)	Anion binding	6	2.64 × 10^-7^	3.43 × 10^-6^
GO:0005230 (MF)	Extracellular ligand-gated ion channel activity	5	2.22 × 10^-6^	2.57 × 10^-5^
GO:0008509 (MF)	Anion transmembrane transporter activity	6	3.99 × 10^-6^	4.15 × 10^-5^
GO:0045211 (CC)	Postsynaptic membrane	8	2.22 × 10^-9^	1.86 × 10^-7^
GO:0034707 (CC)	Chloride channel complex	6	6.57 × 10^-9^	2.76 × 10^-7^
GO:0044456 (CC)	Synapse part	9	1.20 × 10^-8^	3.36 × 10^-7^
GO:0045202 (CC)	Synapse	9	2.51 × 10^-7^	5.27 × 10^-6^
GO:0030054 (CC)	Cell junction	9	5.88 × 10^-6^	9.88 × 10^-5^
GO:0034702 (CC)	Ion channel complex	6	1.53 × 10^-5^	0.0002
GO:0044459 (CC)	Plasma membrane part	16	1.70 × 10^-5^	0.0002
GO:0031226 (CC)	Intrinsic to plasma membrane	11	0.0002	0.0019
GO:0005887 (CC)	Integral to plasma membrane	11	0.0002	0.0019
GO:0033267 (CC)	Axon part	3	0.0003	0.0025

* BP: biological process; MF: molecular function; and CC: cellular component.

** BH: p-value corrected by the Benjamini-Hochberg method (1995) [50].

To further investigate whether our approach to selecting the 47 genes is efficient, we compared the results with a similar analysis of top-ranked genes based on p values in COGA GWAS. We assigned the smallest p value of the marker mapped to a gene to represent gene-wise association significance. Then, we selected the most significant 47 genes. No functional term was significant in GO term analysis. Of note, our results were not corrected for gene length bias, a potential problem in gene-based association studies [25]. This comparison suggested that our cross-species gene ranking method may be more useful in extracting biological meaning from gene lists.

We further examined the function of the 47 genes selected by cross-species ranking by using the ToppFun online tool [26]. ToppFun provides enrichment analysis of candidate genes in many biological categories, including GO terms, biological pathways, human and mouse phenotypes, protein domains, and reference search in PubMed. We presented the results of ToppFun as complementary information for WebGestalt analysis and summarized the results of enriched pathways in Table 7 enriched mouse phenotypes in Table 8 and enriched PubMed citations in Table 9. In the pathway analysis, ToppFun uses a comprehensive collection of pathways from major databases such as KEGG, Reactome, and BioCarta [26]. The most enriched pathway is neuroactive ligand-receptor interaction (p = 5.36 × 10^-5^). Other significant pathways included GPCR ligand binding and G alpha signaling events; here, GPCR denotes G protein-coupled receptor (Table 7). Moreover, mouse phenotype analysis revealed that our selected genes are involved in neuron-related activity (Table 8). Overall, pathway and mouse phenotype enrichment analyses confirmed the results obtained from the GO term enrichment analysis by WebGestalt, and these analyses also revealed highlighted genes related to synaptic activity and GABA signaling as being particularly represented in significant pathways and mouse phenotypes. Finally, we queried PubMed references by ToppFun to search for publications that are overrepresented with genes from our top ranked list (Table 9). The highest scored record from this analysis was from a genetic study of gene expression associated with alcohol consumption in rats and humans [4], in which 24 of our top genes were represented in the total of 130 genes described by this study and showed significant enrichment (p < 1 × 10^-6^). The second highest scored record was from an association study of 182 candidate genes in anorexia nervosa (enrichment p < 1 × 10^-6^).

**Table 7:** Pathways significantly associated with top candidate genes by ToppFun.

Pathway ID/name	Description	Source	p-value	Terms in query	Terms in genome
hsa04080	Neuroactive ligand-receptor interaction	KEGG pathway	5.36 × 10^-5^	10	256
REACTOME_GPCR_LIGAND_BINDING	Genes involved in GPCR ligand binding	MSigDB: C2.cp - Reactome	2.66 × 10^-3^	10	392
REACTOME_PEPTIDE_LIGAND_BINDING_RECEPTORS	Genes involved in Peptide ligand-binding receptors	MSigDB: C2.cp - Reactome	4.36 × 10^-3^	7	173
REACTOME_DOWNSTREAM_EVENTS_IN_GPCR_SIGNALING	Genes involved in Downstream events in GPCR signaling	MSigDB: C2.cp - Reactome	8.63 × 10^-3^	10	448
REACTOME_CLASS_A1_RHODOPSIN_LIKE_RECEPTORS	Genes involved in Class A/1 (Rhodopsin-like receptors)	MSigDB: C2.cp - Reactome	1.62 × 10^-2^	8	292
REACTOME_G_ALPHA_Q_SIGNALLING_EVENTS	Genes involved in G alpha (q) signaling events	MSigDB: C2.cp - Reactome	2.78 × 10^-2^	6	157

**Table 8:** Mouse phenotypes significantly associated with top candidate genes by ToppFun.

Phenotype ID	Phenotype name	p-value	Terms in query	Terms in genome
MP:0009745	Abnormal behavioral response to xenobiotic	6.77 × 10^-7^	12	215
MP:0002206	Abnormal CNS synaptic transmission	4.31 × 10^-6^	14	382
MP:0003635	Abnormal synaptic transmission	3.57 × 10^-5^	14	450
MP:0002062	Abnormal conditioning behavior	6.87 × 10^-5^	10	199
MP:0002063	Abnormal learning/memory/conditioning	9.26 × 10^-5^	13	405
MP:0002065	Abnormal fear/anxiety-related behavior	1.05 × 10^-4^	10	208
MP:0001362	Abnormal anxiety-related response	3.59 × 10^-4^	9	179
MP:0002572	Abnormal emotion/affect behavior	8.34 × 10^-4^	11	329
MP:0001454	Abnormal cued conditioning behavior	1.41 × 10^-3^	6	66
MP:0003633	Abnormal nervous system physiology	2.44 × 10^-3^	20	1333
MP:0001399	Hyperactivity	2.57 × 10^-3^	9	226
MP:0001363	Increased anxiety-related response	2.86 × 10^-3^	7	117
MP:0009357	Abnormal seizure response to inducing agent	9.04 × 10^-3^	7	139
MP:0001449	Abnormal learning/memory	1.22 × 10^-2^	10	349
MP:0003088	Abnormal prepulse inhibition	1.45 × 10^-2^	5	57
MP:0003313	Abnormal locomotor activation	1.46 × 10^-2^	13	630
MP:0000950	abnormal seizure response to pharmacological agent	1.53 × 10^-2^	6	99
MP:0002945	Abnormal inhibitory postsynaptic currents	1.58 × 10^-2^	5	58
MP:0004008	Abnormal GABA-mediated receptor currents	2.85 × 10^-2^	3	11
MP:0009747	Impaired behavioral response to xenobiotic	3.45 × 10^-2^	5	68
MP:0004747	Abnormal cochlear OHC afferent innervation pattern	3.48 × 10^-2^	2	2

**Table 9:** PubMed citations significantly over-represented with top candidate genes by ToppFun.

PubMed ID	Description	p-value	Terms in query	Terms in publication
19874574	Genetical genomic determinants of alcohol consumption in rats and humans.	< 1 × 10^-6^	24	130
20468064	Association study of 182 candidate genes in anorexia nervosa.	< 1 × 10^-6^	15	182
18985723	GABA neurotransmitter signaling in the developing mouse lens: dynamic regulation of components and functionality.	< 1 × 10^-6^	7	18
21205893	TrkB receptor controls striatal formation by regulating the number of newborn striatal neurons.	< 1 × 10^-6^	6	12
16987237	Reduced expression of neuropeptide genes in a genome-wide screen of a secretion-deficient mouse.	< 1 × 10^-6^	8	67

## Discussion

In this work, we applied a unique cross-species, evidence-based gene prioritization strategy for genes involved in alcoholism. We started with a set of genes with prior microarray expression evidence of involvement in ethanol response, representing approximately 10% of the human protein-coding genes. These genes were ranked using additional sources of evidence across multiple species, including humans, mice, *C. elegans *and *Drosophila*. We used the COGA GWAS dataset and applied permutation analysis to evaluate the best weighting score matrix for gene ranking. Based on these results, we selected the top 47 genes with the best evidence for follow up bioinformatics analysis. Our functional enrichment test of these 47 genes suggested that this ranking algorithm identifies gene sets with coherent biological functions relevant to brain responses to ethanol and neural adaptations occurring with alcoholism. Remarkably, higher ranking scores were predictive of genes containing an enrichment of significant SNP associations in the context of COGA alcohol dependence GWAS results. These results provide initial evidence that a cross-species analysis of gene networks correlated with molecular or behavioral responses to ethanol may provide a powerful strategy to identify candidate genes that contribute to alcoholism.

The identification of genes mediating biological responses to ethanol, including the modification of risk profiles for alcoholism, is an area of intense research interest due to the possibility of pinpointing targets for future alcoholism therapies. Recent advances in behavioral genetics and genomics have identified large numbers of genes that potentially contribute to phenotypic responses to ethanol in both human and animal models. However, little progress has been made in narrowing or organizing these large lists of genes into a tractable scheme for understanding the neurobiology and genetics of alcoholism. One approach that has been used for large collections of microarray data has been the performance of a meta-analysis across data on rodent models of divergent ethanol drinking collected from multiple centers and strains [3]. However, this analysis identified 3,800 genes associated with variation in ethanol intake, making downstream hypothesis-driven studies difficult to formulate.

As discussed in the Background, in our research approach, we pursued a gene ranking algorithm constructed to integrate data on ethanol-related genes across species. We recognized that direct behavioral parallels with ethanol response across humans, mice, *Drosophila *and *C. elegans *were likely to be tenuous or non-existent. However, molecular commonalities underlying ethanol responses across species, if they could be identified, should provide a powerful validation mechanism for candidate genes involved in ethanol behavioral responses, even if those particular behavioral components differ across species.

Our ranking algorithm, while largely empirical at this stage, identified a ranked list of genes with obvious coherence in terms of functional gene networks. A remarkably large number of genes already validated as altering behavioral responses to ethanol were contained in the higher ranks. In addition, bioinformatics analysis showed several interesting biological functions that were over-represented among the ranked genes (Tables 6, 7, 8, 9), which is largely consistent with our previous analysis based on a network approach [27]. Again, a number of individual gene members from the constructed networks have strong prior validation as candidate genes that influence alcoholism traits in humans or behavioral responses to ethanol in animal models. These validated genes serve to increase the probability for the entire gene network playing a role in ethanol responses.

Although gene targeting approaches in animal models might ultimately be the most robust method for validating the role of individual genes in ethanol response behaviors, such studies are complex and time-consuming. We chose, as an initial approach to validate our cross-species ranking algorithm, a study of the association of the gene ranking score with alcoholism traits in a GWAS analysis. We found a reduction in the minimum FDR q-value as the ranking score increased to 2. It is important to note that this effect is not due to the progressive limiting of markers examined. In this study, FDR is not dependent on the number of tests performed.

Although the results are encouraging, the limitations of the current analysis and possible improvements must be noted. We noted that when the gene rank score cutoff increased from 2.0 to 2.5, the size of the q-values reversed. This observation might be attributed to overly restricted gene selection given that number of SNPs in genes dropped from 2293 in 47 genes to 210 in only 6 genes. Another limitation is that the use of genes from the addiction/alcoholism array represents hypotheses about important genes, as selected by expert review, rather than selection from empirical association data. We could improve the current approach in the following ways. First, although we included seven datasets in the gene ranking, many additional datasets now exist or will be released in the near future that may be used in multi-species data integration. Additionally, this single GWAS dataset is likely to be underpowered given the recent evidence showing many loci of small effect influence most complex human traits. However, a network or pathway analysis approach to analyze a set of genes might improve power [12].

While there are undoubtedly numerous ways to score or weight genes, we have shown that this simple empirical approach is effective. Our results demonstrate the utility of gene ranking after cross-species data integration. Since this initial study demonstrated the utility of this approach, we are continuing to expand the number of data sets and improve the scoring scheme through a more sophisticated optimization of weighting parameters. As more data is included, additional alcohol GWAS results become available, and more sophisticated gene ranking algorithms are developed, we expect improvement in specificity and sensitivity. For example, there are many gene expression studies in rat brain from animals evaluated for alcohol-preference behavior [2,28-31], and they will be integrated in future gene ranking. However, our initial gene targeting experiments in animal models, using the ranked gene lists derived in this study, have already identified several novel genes that alter ethanol response behaviors in mice, *Drosophila *or *C. elegans *(unpublished data). This provides direct support of our cross-species gene ranking.

## Conclusion

In this study, we proposed a cross-species, evidence-based gene ranking strategy and demonstrated it in the eight alcoholism or ethanol response related datasets from four species (human, mouse, fly, and worm). Through the use of permutation and FDR analyses, we evaluated 10 weighting score matrices and found that one of them had the best performance. Using this optimal weighting matrix, we selected 47 genes whose scores were greater than 2 for follow up bioinformatics analysis. Functional enrichment tests revealed that these 47 genes are involved in brain responses to ethanol and neural adaptations occurring with alcoholism. Our results, with further experimental validation in three animal models, suggest that our approach is useful for cross-species gene prioritization.

## Materials and methods

### Cross species gene ranking

In an effort to populate an inclusive gene list with non-biased data from at least two species, we used published microarray gene expression data from our own and other laboratories. As shown in Figure 1, microarray gene expression data was used from three sources: acute responses to ethanol in C57BL/6 and DBA2/J mice (whole genome analysis of samples from reference [14]) that had been supplemented with additional microarray studies (U74B and U74C arrays, Affymetrix) on the same samples, a meta-analysis of genes involved in ethanol preference drinking across multiple mouse strains [3], and analysis of prefrontal cortex from autopsied samples of alcoholic and non-alcoholic brains [15]. We then merged these datasets by utilizing gene homology mapping features within the WebGestalt [32]. This produced a list of 2458 genes. These genes were ranked by scores resulting from the following algorithm:

$$S={\text{w}}_{1}\left(\text{MuAc}\right)+{\text{w}}_{2}\left(\text{MuPref}\right)+{\text{w}}_{3}\left(\text{HuAlc}\right)+{\text{w}}_{4}\left(\text{HuAddChip}\right)+{\text{w}}_{5}\left(\text{HuLink}\right)+{\text{w}}_{6}\left(\text{Ce}\right)+{\text{w}}_{7}\left(\text{Dr}\right)+{\text{w}}_{8}\left(\text{Cross}\right).$$

The symbols refer to sources diagramed in Figure 1. MuAc, MuPref and HuAlc refer to presence in the microarray studies mentioned above. HuAddChip are selected genes from human association studies on alcohol dependence using the "addiction chip" designed by David Goldman and colleagues at the National Institute on Alcohol Abuse and Alcoholism (NIAAA) [19]. HuLink refers to genes contained within linkage regions that have been implicated multiple times across human studies of alcohol-related phenotypes on chromosomes 1, 4, and 7 [16-18]. The region on chromosome 1 ranges from D1S1613 at 64,007,000 bp to D1S2624 at 154,898,000 bp (according to HapMap build 36) and encompasses a variety of overlapping linkage signals to alcohol-related phenotypes, including alcohol dependence, heavy drinking, sensitivity to alcohol, and tolerance, across a number of samples [17,18,33-38]. The chromosome 4 region ranges from D4S2382 at 39,727,200 bp to D4S1615 at 128,429,200 bp and encompasses linkage peaks from four independent samples [16,18,39-42]. The chromosome 7 region ranged from D7S691 at 41,996,200 bp to D7S1817 at 109,026,000 bp and constitutes the strongest linkage region in the Collaborative Study of the Genetics of Alcoholism (COGA) sample [18,43-45]. The invertebrate gene sets are from published studies in *C. elegans *[22] and *Drosophila *[20,21]. Finally, the "Cross" term is a bonus score added for cross-species hits for a given gene except for genes from human linkage regions. The weighting terms w*_i _*(*i *= 1, 2, 3, ... 8) were empirically chosen with 0.5 or 1.0 in 10 different weighting score matrices (Table 1). After a permutation test with COGA GWAS data, we found the weighting score matrix 3 could provide the best performance.

### Analysis of COGA alcohol dependence GWAS dataset

The COGA GWAS dataset was used to evaluate the gene rankings. It contains 1205 cases and 700 controls [23]. All cases met DSM-IV criteria for alcohol dependence. Controls were defined as individuals who have consumed alcohol, but did not meet any definition of alcohol dependence or alcohol abuse, nor did they meet any DSM-IIIR or DSM-IV definition of abuse or dependence for other drugs (except nicotine). The Illumina human 1M chipset was used for genotyping. Only DNA samples achieving a call rate of > 95% were included. A total of 1,041,465 SNP markers were used for case-control analyses. We conducted population stratification and association analyses using PLINK, a highly flexible, fast, and user-friendly package for GWAS analysis [46]. In our analyses we included only SNPs if their genome-wide failure rate did not exceed 0.05. SNPs were further excluded if minor allele frequency was less than 0.01. After these data filtering processes, 958,380 SNPs were used for further analyses. Then, we mapped these SNPs to non-pseudogenes in the NCBI Entrez Gene database. Specifically, a SNP belongs to a gene if it locates in the region within 10 kb upstream to 10 kb downstream of the gene.

### FDR control

To control the risk of false discoveries in GWAS studies, for each p-value, we calculated a *q-value *[47,48]. A q-value is an estimate of the proportion of false discoveries among all significant markers (i.e., q-values are FDRs) when the corresponding p-value is used as the threshold to declare significance. As argued previously [49], we preferred this approach to more traditional multiple testing methods that control the probability of producing one or more false discoveries for a set of tests [50]. Our approach was preferred because these q-values 1) provide a better balance between the competing goals of finding true positives versus controlling false discoveries, 2) allow the use of more similar standards in terms of the proportion of false discoveries produced across studies due to much less dependence on the number of tests performed, 3) are relatively robust against the effects of correlated tests [47,49,51-56], and 4) rather than an all-or-nothing conclusion about whether a study produces significant results, instead provide a more subtle picture about the possible relevance of the tested markers. The FDR procedure is performed in the R statistical package.

### Random permutation for different score matrix

To test the significance of the gene ranking enrichment result for each weighting score matrix, we did 100 random permutations for the q-value enrichment. Since longer genes tend to have more SNPs in GWAS data, to reduce this gene length bias, we restricted the gene length of the random selections in each permutation within ± 50 kb of the average length of our ranked genes. We set the permutation p-value as the proportion of permutation times in which there are higher q-value proportions in randomly selected genes than in our ranked genes in the corresponding score region. For example, there were *n *genes with a score *s *under a specific weighting score matrix. Then, in each permutation, *n *genes were selected from all human genes whose length is ± 50 kb of the average length of the *n *ranked genes. The q-values for SNPs in the randomly selected genes were calculated based on the GWAS data. For simplicity of comparison, we compared the number of SNPs in each q-value range (e.g., < 0.9, < 0.8, etc.). The proportion of the q-value number for each q-value range in randomly selected genes was then calculated. If the proportion was larger than our ranked alcohol genes at the same q-value range, we counted this permutation as a "significant permutation" for the specific score range *s *and q-value range. After 100 permutations, the proportion of "significant permutation" was set as the p-value of our permutation result at the corresponding score and q-value range. For the weighting score matrix with the best performance, permutation testing was repeated 1000 times again to check the significance.

### Bioinformatics analysis of cross-species ranked gene list

The 47 top ranked genes with a score ≥ 2 were examined for enriched GO terms using the WebGestalt online tool (version 2) [32]. This tool examines the over-representation of genes of interest in GO terms based on the hypergeometric test followed by the Benjamini-Hochberg (1995) adjustment of p-values [50]. We then used the ToppFun online tool [57], which is an integrated over-representation analysis tool, to interrogate databases for biological pathways, mouse phenotypes, and PubMed citations.

## Competing interests

The authors declare that they have no competing interests.

## Authors' contributions

AYG FA HJE DMD MAS AA JK and JIN collected and prepared data for this study. AYG, FA, BTW, ZZ, PJ, and MFM conducted data analysis. ZZ, EJO, BPR, DMD, JCB, AGD, MSG, KSK, BTW and MFM conceived and designed the study. ZZ, AYG, BTW, MFM, PJ, HJE, MAS, AA, JK, and JIN contributed to the writing of the manuscript. All authors read and approved the final manuscript.

## Supplementary Material

Additional file 1 **Summary of the number and proportion of q-values, and p-value of 100 permutation results for different ranked score and q-value range under each of the 10 weighting score matrices**. Click here for file

## References

[B1] Guo AY Webb BT Miles MF Zimmerman MP Kendler KS Zhao Z ERGR: An ethanol-related gene resource Nucleic Acids Res 2009 37 D840 845 10.1093/nar/gkn816 18978021 PMC2686553

[B2] Kimpel MW Strother WN McClintick JN Carr LG Liang T Edenberg HJ McBride WJ Functional gene expression differences between inbred alcohol-preferring and -non-preferring rats in five brain regions Alcohol 2007 41 95 132 10.1016/j.alcohol.2007.03.003 17517326 PMC1976291

[B3] Mulligan MK Ponomarev I Hitzemann RJ Belknap JK Tabakoff B Harris RA Crabbe JC Blednov YA Grahame NJ Phillips TJ Toward understanding the genetics of alcohol drinking through transcriptome meta-analysis Proc Natl Acad Sci USA 2006 103 6368 6373 10.1073/pnas.0510188103 16618939 PMC1458884

[B4] Tabakoff B Saba L Printz M Flodman P Hodgkinson C Goldman D Koob G Richardson HN Kechris K Bell RL Genetical genomic determinants of alcohol consumption in rats and humans BMC Biol 2009 7 70 10.1186/1741-7007-7-70 19874574 PMC2777866

[B5] Sun J Han L Zhao Z Gene- and evidence-based candidate gene selection for schizophrenia and gene feature analysis Artif Intell Med 2010 48 99 106 10.1016/j.artmed.2009.07.009 19944577 PMC2826526

[B6] Sun J Jia P Fanous AH Webb BT van den Oord EJ Chen X Bukszar J Kendler KS Zhao Z A multi-dimensional evidence-based candidate gene prioritization approach for complex diseases-schizophrenia as a case Bioinformatics 2009 25 2595 2602 10.1093/bioinformatics/btp428 19602527 PMC2752609

[B7] Rodd ZA Bertsch BA Strother WN Le-Niculescu H Balaraman Y Hayden E Jerome RE Lumeng L Nurnberger JI Edenberg HJ Candidate genes, pathways and mechanisms for alcoholism: an expanded convergent functional genomics approach Pharmacogenomics J 2007 7 222 256 10.1038/sj.tpj.6500420 17033615

[B8] Huang HC Zheng S VanBuren V Zhao Z Discovering disease-specific biomarker genes for cancer diagnosis and prognosis Technol Cancer Res Treat 2010 9 219 230 20441232 10.1177/153303461000900301

[B9] Jia P Sun J Guo A Zhao Z SZGR: a comprehensive schizophrenia gene resource Mol Psychiatry 2010 15 453 462 10.1038/mp.2009.93 20424623 PMC2861797

[B10] Sun J Kuo PH Riley BP Kendler KS Zhao Z Candidate genes for schizophrenia: a survey of association studies and gene ranking Am J Med Genet B Neuropsychiatr Genet 2008 147B 1173 1181 10.1002/ajmg.b.30743 18361404

[B11] Kendler KS Schizophrenia genetics and dysbindin: a corner turned? Am J Psychiatry 2004 161 1533 1536 10.1176/appi.ajp.161.9.1533 15337639

[B12] Jia P Wang L Meltzer HY Zhao Z Pathway-based analysis of GWAS datasets: effective but caution required Int J Neuropsychopharmacol 2011 14 567 572 10.1017/S1461145710001446 21208483

[B13] Schuckit MA Low level of response to alcohol as a predictor of future alcoholism Am J Psychiatry 1994 151 184 189 8296886 10.1176/ajp.151.2.184

[B14] Kerns RT Ravindranathan A Hassan S Cage MP York T Sikela JM Williams RW Miles MF Ethanol-responsive brain region expression networks: implications for behavioral responses to acute ethanol in DBA/2J versus C57BL/6J mice J Neurosci 2005 25 2255 2266 10.1523/JNEUROSCI.4372-04.2005 15745951 PMC6726093

[B15] Mayfield RD Lewohl JM Dodd PR Herlihy A Liu J Harris RA Patterns of gene expression are altered in the frontal and motor cortices of human alcoholics J Neurochem 2002 81 802 813 10.1046/j.1471-4159.2002.00860.x 12065639

[B16] Prescott CA Sullivan PF Kuo PH Webb BT Vittum J Patterson DG Thiselton DL Myers JM Devitt M Halberstadt LJ Genomewide linkage study in the Irish affected sib pair study of alcohol dependence: evidence for a susceptibility region for symptoms of alcohol dependence on chromosome 4 Mol Psychiatry 2006 11 603 611 10.1038/sj.mp.4001811 16534506

[B17] Kuo PH Neale MC Riley BP Webb BT Sullivan PF Vittum J Patterson DG Thiselton DL van den Oord EJ Walsh D Identification of susceptibility loci for alcohol-related traits in the Irish Affected Sib Pair Study of Alcohol Dependence Alcohol Clin Exp Res 2006 30 1807 1816 10.1111/j.1530-0277.2006.00217.x 17067344

[B18] Reich T Edenberg HJ Goate A Williams JT Rice JP Van Eerdewegh P Foroud T Hesselbrock V Schuckit MA Bucholz K Genome-wide search for genes affecting the risk for alcohol dependence Am J Med Genet 1998 81 207 215 10.1002/(SICI)1096-8628(19980508)81:3<207::AID-AJMG1>3.0.CO;2-T 9603606

[B19] Hodgkinson CA Yuan Q Xu K Shen PH Heinz E Lobos EA Binder EB Cubells J Ehlers CL Gelernter J Addictions biology: haplotype-based analysis for 130 candidate genes on a single array Alcohol Alcohol 2008 43 505 515 18477577 10.1093/alcalc/agn032 PMC2724863

[B20] Morozova TV Anholt RR Mackay TF Phenotypic and transcriptional response to selection for alcohol sensitivity in Drosophila melanogaster Genome Biol 2007 8 R231 10.1186/gb-2007-8-10-r231 17973985 PMC2246305

[B21] Morozova TV Anholt RR Mackay TF Transcriptional response to alcohol exposure in Drosophila melanogaster Genome Biol 2006 7 R95 10.1186/gb-2006-7-10-r95 17054780 PMC1794562

[B22] Kwon JY Hong M Choi MS Kang S Duke K Kim S Lee S Lee J Ethanol-response genes and their regulation analyzed by a microarray and comparative genomic approach in the nematode Caenorhabditis elegans Genomics 2004 83 600 614 10.1016/j.ygeno.2003.10.008 15028283

[B23] Edenberg HJ Koller DL Xuei X Wetherill L McClintick JN Almasy L Bierut LJ Bucholz KK Goate A Aliev F Genome-wide association study of alcohol dependence implicates a region on chromosome 11 Alcohol Clin Exp Res 2010 34 840 852 10.1111/j.1530-0277.2010.01156.x 20201924 PMC2884073

[B24] Dick DM Rose RJ Kaprio J The next challenge for psychiatric genetics: characterizing the risk associated with identified genes Ann Clin Psychiatry 2006 18 223 231 10.1080/10401230600948407 17162621 PMC1764634

[B25] Jia P Tian J Zhao Z Assessing gene length biases in gene set analysis of Genome-Wide Association Studies Int J Comput Biol Drug Des 2010 3 297 310 10.1504/IJCBDD.2010.038394 21297229

[B26] Chen J Bardes EE Aronow BJ Jegga AG ToppGene Suite for gene list enrichment analysis and candidate gene prioritization Nucleic Acids Res 2009 37 W305 311 10.1093/nar/gkp427 19465376 PMC2703978

[B27] Guo AY Sun J Jia P Zhao Z Network analysis of ethanol-related candidate genes Chem Biodiv 2010 7 1142 1152 10.1002/cbdv.200900318 PMC2935470 20491071

[B28] Edenberg HJ Strother WN McClintick JN Tian H Stephens M Jerome RE Lumeng L Li TK McBride WJ Gene expression in the hippocampus of inbred alcohol-preferring and -nonpreferring rats Genes Brain Behav 2005 4 20 30 15660665 10.1111/j.1601-183X.2004.00091.x

[B29] Rodd ZA Kimpel MW Edenberg HJ Bell RL Strother WN McClintick JN Carr LG Liang T McBride WJ Differential gene expression in the nucleus accumbens with ethanol self-administration in inbred alcohol-preferring rats Pharmacol Biochem Behav 2008 89 481 498 10.1016/j.pbb.2008.01.023 18405950 PMC4516281

[B30] Carr LG Kimpel MW Liang T McClintick JN McCall K Morse M Edenberg HJ Identification of candidate genes for alcohol preference by expression profiling of congenic rat strains Alcohol Clin Exp Res 2007 31 1089 1098 10.1111/j.1530-0277.2007.00397.x 17451403 PMC4455872

[B31] Liang T Kimpel MW McClintick JN Skillman AR McCall K Edenberg HJ Carr LG Candidate genes for alcohol preference identified by expression profiling in alcohol-preferring and -nonpreferring reciprocal congenic rats Genome Biol 2010 11 R11 10.1186/gb-2010-11-2-r11 20128895 PMC2872871

[B32] WebGestal http://bioinfo.vanderbilt.edu/webgestalt/

[B33] Dick DM Nurnberger J Edenberg HJ Goate A Crowe R Rice J Bucholz KK Kramer J Schuckit MA Smith TL Suggestive linkage on chromosome 1 for a quantitative alcohol-related phenotype Alcohol Clin Exp Res 2002 26 1453 1460 10.1111/j.1530-0277.2002.tb02443.x 12394277

[B34] Guerrini I Cook CC Kest W Devitgh A McQuillin A Curtis D Gurling HM Genetic linkage analysis supports the presence of two susceptibility loci for alcoholism and heavy drinking on chromosome 1p22.1-11.2 and 1q21.3-24.2 BMC Genet 2005 6 11 15740611 10.1186/1471-2156-6-11 PMC554783

[B35] Hill SY Shen S Zezza N Hoffman EK Perlin M Allan W A genome wide search for alcoholism susceptibility genes Am J Med Genet B Neuropsychiatr Genet 2004 128B 102 113 10.1002/ajmg.b.30013 15211641 PMC3285396

[B36] Lappalainen J Long JC Eggert M Ozaki N Robin RW Brown GL Naukkarinen H Virkkunen M Linnoila M Goldman D Linkage of antisocial alcoholism to the serotonin 5-HT1B receptor gene in 2 populations Arch Gen Psychiatry 1998 55 989 994 10.1001/archpsyc.55.11.989 9819067

[B37] Nurnberger JI Foroud T Flury L Su J Meyer ET Hu K Crowe R Edenberg H Goate A Bierut L Evidence for a locus on chromosome 1 that influences vulnerability to alcoholism and affective disorder Am J Psychiatry 2001 158 718 724 10.1176/appi.ajp.158.5.718 11329392

[B38] Schuckit MA Edenberg HJ Kalmijn J Flury L Smith TL Reich T Bierut L Goate A Foroud T A genome-wide search for genes that relate to a low level of response to alcohol Alcohol Clin Exp Res 2001 25 323 329 10.1111/j.1530-0277.2001.tb02217.x 11290841

[B39] Ehlers CL Gilder DA Wall TL Phillips E Feiler H Wilhelmsen KC Genomic screen for loci associated with alcohol dependence in Mission Indians Am J Med Genet B Neuropsychiatr Genet 2004 129B 110 115 10.1002/ajmg.b.30057 15274051

[B40] Long JC Knowler WC Hanson RL Robin RW Urbanek M Moore E Bennett PH Goldman D Evidence for genetic linkage to alcohol dependence on chromosomes 4 and 11 from an autosome-wide scan in an American Indian population Am J Med Genet 1998 81 216 221 10.1002/(SICI)1096-8628(19980508)81:3<216::AID-AJMG2>3.0.CO;2-U 9603607

[B41] Saccone NL Kwon JM Corbett J Goate A Rochberg N Edenberg HJ Foroud T Li TK Begleiter H Reich T Rice JP A genome screen of maximum number of drinks as an alcoholism phenotype Am J Med Genet 2000 96 632 637 10.1002/1096-8628(20001009)96:5<632::AID-AJMG8>3.0.CO;2-# 11054770

[B42] Williams JT Begleiter H Porjesz B Edenberg HJ Foroud T Reich T Goate A Van Eerdewegh P Almasy L Blangero J Joint multipoint linkage analysis of multivariate qualitative and quantitative traits. II. Alcoholism and event-related potentials Am J Hum Genet 1999 65 1148 1160 10.1086/302571 10486334 PMC1288248

[B43] Dunn G Hinrichs AL Bertelsen S Jin CH Kauwe JS Suarez BK Bierut LJ Microsatellites versus single-nucleotide polymorphisms in linkage analysis for quantitative and qualitative measures BMC Genet 2005 6 Suppl 1 S122 10.1186/1471-2156-6-S1-S122 16451580 PMC1866815

[B44] Foroud T Edenberg HJ Goate A Rice J Flury L Koller DL Bierut LJ Conneally PM Nurnberger JI Bucholz KK Alcoholism susceptibility loci: confirmation studies in a replicate sample and further mapping Alcohol Clin Exp Res 2000 24 933 945 10.1111/j.1530-0277.2000.tb04634.x 10923994

[B45] Wang JC Hinrichs AL Stock H Budde J Allen R Bertelsen S Kwon JM Wu W Dick DM Rice J Evidence of common and specific genetic effects: association of the muscarinic acetylcholine receptor M2 (CHRM2) gene with alcohol dependence and major depressive syndrome Hum Mol Genet 2004 13 1903 1911 10.1093/hmg/ddh194 15229186

[B46] Purcell S Neale B Todd-Brown K Thomas L Ferreira MA Bender D Maller J Sklar P de Bakker PI Daly MJ Sham PC PLINK: a tool set for whole-genome association and population-based linkage analyses Am J Hum Genet 2007 81 559 575 10.1086/519795 17701901 PMC1950838

[B47] Storey JD The positive false discovery rate: A Bayesian interpretation and the q-value Annals Stat 2003 31 2013 2035 10.1214/aos/1074290335

[B48] Storey JD Tibshirani R Statistical significance for genomewide studies Proc Natl Acad Sci USA 2003 100 9440 9445 10.1073/pnas.1530509100 12883005 PMC170937

[B49] van den Oord EJ Sullivan PF False discoveries and models for gene discovery Trends Genet 2003 19 537 542 10.1016/j.tig.2003.08.003 14550627

[B50] Benjamini Y Hochberg Y Controlling the false discovery rate: a practical and powerful approach to multiple testing J R Statist Soc B 1995 289 300

[B51] Brown BW Russell K Methods of correcting for multiple testing: operating characteristics Stat Med 1997 16 2511 2528 10.1002/(SICI)1097-0258(19971130)16:22<2511::AID-SIM693>3.0.CO;2-4 9403953

[B52] Fernando RL Nettleton D Southey BR Dekkers JC Rothschild MF Soller M Controlling the proportion of false positives in multiple dependent tests Genetics 2004 166 611 619 10.1534/genetics.166.1.611 15020448 PMC1470704

[B53] Korn EL Troendle JF McShane LM Simon R Controlling the number of false discoveries: application to high-dimensional genomic data J Stat Plan Infer 2004 124 379 398 10.1016/S0378-3758(03)00211-8

[B54] Sabatti C Service S Freimer N False discovery rate in linkage and association genome screens for complex disorders Genetics 2003 164 829 833 12807801 10.1093/genetics/164.2.829 PMC1462572

[B55] Tsai CA Hsueh HM Chen JJ Estimation of false discovery rates in multiple testing: application to gene microarray data Biometrics 2003 59 1071 1081 10.1111/j.0006-341X.2003.00123.x 14969487

[B56] van den Oord EJ Controlling false discoveries in candidate gene studies Mol Psychiatry 2005 10 230 231 10.1038/sj.mp.4001581 15738930

[B57] ToppFun http://toppgene.cchmc.org/

