# Supplementary material for: Multi-species data integration and gene ranking enrich significant results in an alcoholism genome-wide association study
Source: BMC Genomics. 2012 Dec 17;13(Suppl 8):S16. doi: 10.1186/1471-2164-13-S8-S16 (PMC3535715; doi:10.1186/1471-2164-13-S8-S16)
Supplement: Additional file 1 — Summary of the number and proportion of q-values, and p-value of 100 permutation results for different ranked score and q-value range under each of the 10 weighting score matrices. [file 1471-2164-13-S8-S16-S1.pdf]

Additional file 1 - This additional file summarizes the number and proportion of q-values, and p-value of 100 permutation results for different ranked score and q-value range under each of the 10 weighting score matrices.

Weighting score matrix 1

Table 1. Number and proportion of q-values for each q-value range and ranked gene score under weighting score matrix 1.

Score	≥0.5	≥1	≥1.5	≥2	≥2.5	=3
All q value	91774 (100%)	29001 (100%)	8651 (100%)	7516 (100%)	6539 (100%)	1094 (100%)
q<0.9	6399 (7.0%)	3000 (10.3%)	382 (4.4%)	2012 (26.8%)	710 (10.9%)	161 (14.7%)
q<0.8	469 (0.5%)	592 (2.0%)	151 (1.7%)	148 (2.0%)	144 (2.2%)	0
q<0.7	8 (0.009%)	2 (0.007%)	5 (0.06%)	42 (0.6%)	67 (1.0%)	0
q<0.6	5 (0.005%)	2 (0.007%)	0	0	5 (0.08%)	0
q<0.5	5 (0.005%)	0	0	0	0	0
q<0.4	5 (0.005%)	0	0	0	0	0
q<0.3	0	0	0	0	0	0
q<0.2	0	0	0	0	0	0
q<0.1	0	0	0	0	0	0
q<0.05	0	0	0	0	0	0

Numbers in the parentheses are the proportion of number of q-values.

Table 2. P-value of 100 permutation result for different ranked gene score and q-value range under weighting score matrix 1.

Score	≥0.5	≥1	≥1.5	≥2	≥2.5	=3
q<0.9	0.34	0.31	0.63	0.15	0.44	0.57
q<0.8	0.5	0.32	0.48	0.48	0.49	N/A
q<0.7	0.47	0.68	0.61	0.4	0.37	N/A
q<0.6	0.25	0.5	N/A	N/A	0.5	N/A
q<0.5	0.11	N/A	N/A	N/A	N/A	N/A
q<0.4	0.02	N/A	N/A	N/A	N/A	N/A

N/A: not available due to absence of the real data at those categories.

Weighting score matrix 2

Table 3. Number and proportion of q-values for each q-value range and ranked gene score under weighting score matrix 2.

Score	≥0.5	≥1	≥1.5	≥2	=2.5
All q value	91774 (100%)	29001 (100%)	8651 (100%)	6539 (100%)	1094 (100%)
q<0.9	6399 (7.0%)	3000 (10.3%)	382 (4.4%)	710 (10.9%)	161 (14.7%)
q<0.8	469 (0.5%)	592 (2.0%)	151 (1.7%)	144 (2.2%)	0
q<0.7	8 (0.009%)	2 (0.007%)	5 (0.06%)	67 (1.0%)	0
q<0.6	5 (0.005%)	2 (0.007%)	0	5 (0.08%)	0
q<0.5	5 (0.005%)	0	0	0	0
q<0.4	5 (0.005%)	0	0	0	0
q<0.3	0	0	0	0	0
q<0.2	0	0	0	0	0
q<0.1	0	0	0	0	0
q<0.05	0	0	0	0	0

Table 4. P-value of 100 permutation result for different ranked gene score and q-value range under weighting score matrix 2.

Score	≥0.5	≥1	≥1.5	≥2	=2.5
q<0.9	0.46	0.26	0.68	0.33	0.49
q<0.8	0.47	0.26	0.51	0.35	N/A
q<0.7	0.51	0.59	0.68	0.31	N/A
q<0.6	0.34	0.45	N/A	0.4	N/A
q<0.5	0.17	N/A	N/A	N/A	N/A
q<0.4	0.01	N/A	N/A	N/A	N/A

Weighting score matrix 3

Table 5. Number and proportion of q-values for each q-value range and ranked gene score under weighting score matrix 3.

Score	≥0.5	≥1	≥1.5	≥2	=2.5
All q value	91774 (100%)	18988 (100%)	7948 (100%)	2293 (100%)	210 (100%)
q<0.9	6399 (7.0%)	1863 (9.8%)	399 (5.0%)	866 (37.8%)	178 (84.8%)
q<0.8	469 (0.5%)	415 (2.2%)	163 (2.1%)	199 (8.7%)	108 (51.4%)
q<0.7	8 (0.009%)	164 (0.9%)	5 (0.06%)	117 (5.1%)	72 (34.3%)
q<0.6	5 (0.005%)	0	0	42 (1.8%)	53 (25.2%)
q<0.5	5 (0.005%)	0	0	39 (1.7%)	0
q<0.4	5 (0.005%)	0	0	27 (1.2%)	0
q<0.3	0	0	0	0	0
q<0.2	0	0	0	0	0
q<0.1	0	0	0	0	0
q<0.05	0	0	0	0	0

Table 6. P-value of 100 permutation result for different ranked gene score and q-value range under weighting score matrix 3.

Score	≥0.5	≥1	≥1.5	≥2	=2.5
q<0.9	0.39	0.42	0.68	0.18	0.05
q<0.8	0.49	0.35	0.53	0.28	0.16
q<0.7	0.5	0.23	0.67	0.21	0.09
q<0.6	0.24	N/A	N/A	0.26	0.05
q<0.5	0.07	N/A	N/A	0.16	N/A
q<0.4	0.01	N/A	N/A	0.13	N/A

Weighting score matrix 4

Table 7. Number and proportion of q-values for each q-value range and ranked gene score under weighting score matrix 4.

Score	≥0.5	≥1	≥1.5	≥2	≥2.5	=3
All q value	91774 (100%)	29001 (100%)	15768 (100%)	7674 (100%)	1191 (100%)	570 (100%)
q<0.9	6399 (7.0%)	3000 (10.3%)	692 (4.4%)	349 (4.5%)	2 (0.2%)	114 (20%)
q<0.8	469 (0.5%)	592 (2.0%)	305 (1.9%)	143 (1.9%)	0	76 (13.3%)
q<0.7	8 (0.009%)	2 (0.007%)	86 (0.5%)	5 (0.07%)	0	0
q<0.6	5 (0.005%)	2 (0.007%)	0	0	0	0
q<0.5	5 (0.005%)	0	0	0	0	0
q<0.4	5 (0.005%)	0	0	0	0	0
q<0.3	0	0	0	0	0	0
q<0.2	0	0	0	0	0	0
q<0.1	0	0	0	0	0	0
q<0.05	0	0	0	0	0	0

Table 8. P-value of 100 permutation result for different ranked gene score and q-value range under weighting score matrix 4.

Score	≥0.5	≥1	≥1.5	≥2	≥2.5	=3
q<0.9	0.41	0.36	0.59	0.58	0.83	0.51
q<0.8	0.52	0.29	0.36	0.43	N/A	0.34
q<0.7	0.57	0.63	0.29	0.58	N/A	N/A
q<0.6	0.31	0.4	N/A	N/A	N/A	N/A
q<0.5	0.12	N/A	N/A	N/A	N/A	N/A
q<0.4	0.03	N/A	N/A	N/A	N/A	N/A

Weighting score matrix 5

Table 9. Number and proportion of q-values for each q-value range and ranked gene score under weighting score matrix 5.

Score	≥1	≥1.5	≥2	≥2.5	≥3	=3.5
All q value	91774 (100%)	18988 (100%)	11168 (100%)	6686 (100%)	1171 (100%)	266 (100%)
q<0.9	6399 (7.0%)	1863 (9.8%)	2920 (26.1%)	294 (4.4%)	310 (26.5%)	132 (50.0%)
q<0.8	469 (0.5%)	415 (2.2%)	397 (3.6%)	126 (1.9%)	0	5 (1.9%)
q<0.7	8 (0.009%)	164 (0.9%)	109 (1.0%)	0	0	4 (1.5%)
q<0.6	5 (0.005%)	0	0	0	0	2 (0.8%)
q<0.5	5 (0.005%)	0	0	0	0	0
q<0.4	5 (0.005%)	0	0	0	0	0
q<0.3	0	0	0	0	0	0
q<0.2	0	0	0	0	0	0
q<0.1	0	0	0	0	0	0
q<0.05	0	0	0	0	0	0

Table 10. P-value of 100 permutation result for different ranked gene score and q-value range under weighting score matrix 5.

Score	≥1	≥1.5	≥2	≥2.5	≥3	=3.5
q<0.9	0.42	0.33	0.06	0.64	0.36	0.54
q<0.8	0.59	0.33	0.18	0.44	N/A	0.76
q<0.7	0.58	0.27	0.2	N/A	N/A	0.68
q<0.6	0.29	N/A	N/A	N/A	N/A	0.54
q<0.5	0.12	N/A	N/A	N/A	N/A	N/A
q<0.4	0	N/A	N/A	N/A	N/A	N/A

Weighting score matrix 6

Table 11. Number and proportion of q-values for each q-value range and ranked gene score under weighting score matrix 6.

Score	≥0.5	≥1	≥1.5	≥2	≥2.5	=3
All q value	91774 (100%)	81761 (100%)	18285 (100%)	10883 (100%)	1700 (100%)	405 (100%)
q<0.9	6399 (7.0%)	5600 (6.8%)	1902 (10.4%)	728 (6.7%)	0	224 (55.3%)
q<0.8	469 (0.5%)	463 (0.6%)	507 (2.8%)	272 (2.5%)	0	117 (28.9%)
q<0.7	8 (0.009%)	26 (0.03%)	179 (1.0%)	59 (0.5%)	0	6 (1.5%)
q<0.6	5 (0.005%)	4 (0.005%)	0	0	0	6 (1.5%)
q<0.5	5 (0.005%)	4 (0.005%)	0	0	0	0
q<0.4	5 (0.005%)	0	0	0	0	0
q<0.3	0	0	0	0	0	0
q<0.2	0	0	0	0	0	0
q<0.1	0	0	0	0	0	0
q<0.05	0	0	0	0	0	0

Table 12. P-value of 100 permutation result for different ranked gene score and q-value range under weighting score matrix 6.

Score	≥0.5	≥1	≥1.5	≥2	≥2.5	=3
q<0.9	0.46	0.39	0.38	0.61	N/A	0.29
q<0.8	0.5	0.59	0.23	0.39	N/A	0.26
q<0.7	0.51	0.21	0.24	0.38	N/A	0.57
q<0.6	0.32	0.11	N/A	N/A	N/A	0.46
q<0.5	0.2	0.02	N/A	N/A	N/A	N/A
q<0.4	0.03	N/A	N/A	N/A	N/A	N/A

Weighting score matrix 7

Table 13. Number and proportion of q-values for each q-value range and ranked gene score under weighting score matrix 7.

Score	≥1	≥2	≥2.5	≥3	=3.5
All q value	91774 (100%)	18988 (100%)	6686 (100%)	1762 (100%)	774 (100%)
q<0.9	6399 (7.0%)	1863 (9.8%)	294 (4.4%)	0	2 (0.3%)
q<0.8	469 (0.5%)	415 (2.2%)	126 (1.9%)	0	2 (0.3%)
q<0.7	8 (0.009%)	164 (0.09%)	0	0	0
q<0.6	5 (0.005%)	0	0	0	0
q<0.5	5 (0.005%)	0	0	0	0
q<0.4	5 (0.005%)	0	0	0	0
q<0.3	0	0	0	0	0
q<0.2	0	0	0	0	0
q<0.1	0	0	0	0	0
q<0.05	0	0	0	0	0

Table 14. P-value of 100 permutation result for different ranked gene score and q-value range under weighting score matrix 7.

Score	≥1	≥2	≥2.5	≥3	=3.5
q<0.9	0.4	0.53	0.59	N/A	0.8
q<0.8	0.46	0.33	0.47	N/A	0.65
q<0.7	0.56	0.24	N/A	N/A	N/A
q<0.6	0.33	N/A	N/A	N/A	N/A
q<0.5	0.14	N/A	N/A	N/A	N/A

Weighting score matrix 8

Table 15. Number and proportion of q-values for each q-value range and ranked gene score under weighting score matrix 8.

Score	≥0.5	≥1	≥1.5	≥2	≥2.5	=3
All q value	91774 (100%)	18988 (100%)	7948 (100%)	7516 (100%)	2293 (100%)	210 (100%)
q<0.9	6399 (7.0%)	1863 (9.8%)	399 (5.0%)	2012 (26.7%)	866 (37.8%)	178 (84.8%)
q<0.8	469 (0.5%)	415 (2.2%)	163 (2.1%)	148 (2.0%)	199 (8.7%)	108 (51.4%)
q<0.7	8 (0.009%)	164 (0.09%)	5 (0.06%)	42 (0.6%)	117 (5.1%)	72 (34.3%)
q<0.6	5 (0.005%)	0	0	0	42 (1.8%)	53 (25.2%)
q<0.5	5 (0.005%)	0	0	0	39 (1.7%)	0
q<0.4	5 (0.005%)	0	0	0	27 (1.2%)	0
q<0.3	0	0	0	0	0	0
q<0.2	0	0	0	0	0	0
q<0.1	0	0	0	0	0	0
q<0.05	0	0	0	0	0	0

Table 16. P-value of 100 permutation result for different ranked gene score and q-value range under weighting score matrix 8.

Score	≥0.5	≥1	≥1.5	≥2	≥2.5	=3
q<0.9	0.36	0.4	0.55	0.13	0.2	0.09
q<0.8	0.48	0.34	0.36	0.38	0.24	0.13
q<0.7	0.53	0.29	0.63	0.35	0.16	0.13
q<0.6	0.33	N/A	N/A	N/A	0.21	0.08
q<0.5	0.15	N/A	N/A	N/A	0.1	N/A
q<0.4	0.01	N/A	N/A	N/A	0.08	N/A

Weighting score matrix 9

Table 17. Number and proportion of q-values for each q-value range and ranked gene score under weighting score matrix 9.

Score	≥1	≥2	≥3	=4
All q value	91774 (100%)	18988 (100%)	7948 (100%)	1330 (100%)
q<0.9	6399 (7.0%)	1863 (9.8%)	399 (5.0%)	0
q<0.8	469 (0.5%)	415 (2.2%)	163 (2.1%)	0
q<0.7	8 (0.009%)	164 (0.09%)	5 (0.06%)	0
q<0.6	5 (0.005%)	0	0	0
q<0.5	5 (0.005%)	0	0	0
q<0.4	5 (0.005%)	0	0	0
q<0.3	0	0	0	0
q<0.2	0	0	0	0
q<0.1	0	0	0	0
q<0.05	0	0	0	0

Table 18. P-value of 100 permutation result for different ranked gene score and q-value range under weighting score matrix 9.

Score	≥1	≥2	≥3	=4
q<0.9	0.4	0.36	0.55	N/A
q<0.8	0.49	0.34	0.43	N/A
q<0.7	0.56	0.21	0.6	N/A
q<0.6	0.26	N/A	N/A	N/A
q<0.5	0.08	N/A	N/A	N/A
q<0.4	0.01	N/A	N/A	N/A

Weighting score matrix 10

Table 19. Number and proportion of q-values for each q-value range and ranked gene score under weighting score matrix 10.

Score	≥1	≥2	≥2.5	≥3	=3.5
All q value	91774 (100%)	18988 (100%)	7948 (100%)	1762 (100%)	1330 (100%)
q<0.9	6399 (7.0%)	1863 (9.8%)	399 (5.0%)	0	0
q<0.8	469 (0.5%)	415 (2.2%)	163 (2.1%)	0	0
q<0.7	8 (0.009%)	164 (0.09%)	5 (0.06%)	0	0
q<0.6	5 (0.005%)	0	0	0	0
q<0.5	5 (0.005%)	0	0	0	0
q<0.4	5 (0.005%)	0	0	0	0
q<0.3	0	0	0	0	0
q<0.2	0	0	0	0	0
q<0.1	0	0	0	0	0
q<0.05	0	0	0	0	0

Table 20. P-value of 100 permutation result for different ranked gene score and q-value range under weighting score matrix 10.

Score	≥1	≥2	≥2.5	≥3	=3.5
q<0.9	0.48	0.35	0.65	N/A	N/A
q<0.8	0.56	0.32	0.54	N/A	N/A
q<0.7	0.56	0.24	0.69	N/A	N/A
q<0.6	0.41	N/A	N/A	N/A	N/A
q<0.5	0.18	N/A	N/A	N/A	N/A
q<0.4	0.02	N/A	N/A	N/A	N/A
